# Local versus general anesthesia transperineal prostate biopsy: Tolerability, cancer detection, and complications

**DOI:** 10.1002/bco2.106

**Published:** 2021-09-10

**Authors:** Donnacha Hogan, Abbie Kanagarajah, Henry H. Yao, David Wetherell, Brendan Dias, Phil Dundee, Kevin Chu, Homayoun Zargar, Helen E. O'Connell

**Affiliations:** ^1^ Department of Urology Western Health Melbourne Victoria Australia; ^2^ School of Medicine University College Cork Cork Ireland; ^3^ Melbourne Medical School The University of Melbourne Melbourne Victoria Australia; ^4^ Department of Urology Monash Health Melbourne Victoria Australia

**Keywords:** cancer detection, complications, local anaesthesia, prostate cancer, tolerability, transperineal biopsy

## Abstract

**Objectives:**

To compare data on transperineal template biopsy (TPTB) under general anesthesia (GA) compared with local anesthesia (LA) procedures using the PrecisionPoint™ Transperineal Access System (PPTAS) in relation to tolerability, cancer detection rate, complications, and cost.

**Methods:**

A prospective pilot cohort study of patients undergoing transperineal biopsy was performed. Patients were excluded if they had concurrent flexible cystoscopy or language barriers. Patients had a choice of GA or LA. A prospective questionnaire on Days 0, 1, 7, and 30 was applied. The primary outcome was patient tolerability. Secondary outcomes were cancer detection rate, complication rate, and theater utilization.

**Results:**

This study included 80 patients (40 GA TPTB and 40 LA PPTAS). Baseline characteristics including age, prostate‐specific antigen (PSA), digital rectal examination (DRE), findings, and prostate volume were comparable between the groups (*p* = 0.3790, *p* = 0.9832, *p* = 0.444, *p* = 0.3939, respectively). Higher median prostate imaging‐reporting and data system (PI‐RADS) score of 4 (interquartile range [IQR] 2) versus 3 (IQR 1) was noted in the LA group (*p* = 0.0326). Pain was higher leaving recovery in the GA group however not significantly (*p* = 0.0555). Median pain score at LA infiltration was 5/10 (IQR 3), with no difference in pain at Days 1, 7, or 30 (*p* = 0.2722, 0.6465, and 0.8184, respectively). For GA versus LA, the overall cancer detection rate was 55% versus 55% (*p* = 1.000) with clinically significant cancer in 22.5% versus 35% (*p* = 0.217). Acute urinary retention (AUR) occurred in 5% of GA and 2.5% of LA patients (*p* = 1.000). The GA cohort spent longer in theater and in recovery with a median of 93.5 min versus 57 min for the LA group (*p* = <0.0001).

**Conclusion:**

This study demonstrates that transperineal biopsy is safely performed under LA with no difference between the cohorts in relation cancer detection or AUR. LA biopsy also consumed less theater and recovery resources. A further larger prospective randomized controlled trial is required to confirm the findings of this study.

## 1 INTRODUCTION

There have been several advances in prostate biopsy for the diagnosis of prostate cancer (PCa) in recent years.^1^ Internationally greater than 95% of prostate biopsies were performed using the transrectal route in 2018.^2^ There are several disadvantages of prostate biopsy using the transrectal route. Most importantly, it is associated with a 2%–3% rate of urosepsis which carries significant morbidity and mortality.^3^ Furthermore, studies have demonstrated that transrectal ultrasound guided (TRUS) prostate biopsies perform poorly in the detection of anterior zone lesions and have a lower cancer detection rate than transperineal prostate biopsies (TPBx).^4^ In the last decade, many institutions have changed practice to replace transrectal biopsy with TPBx in a move that has been dubbed by some as “TRexit.”^1^ Currently in the United Kingdom, 32.8% of biopsies are performed via the transperineal route.^5^

Since the introduction of TRUS biopsy, a major concern has been that of infection, specifically severe febrile urinary tract infection (UTI).^3^ This can lead to bacteraemia, urosepsis, septic shock, and multi‐organ dysfunction requiring intensive care support.^3^ An increased prevalence of multiresistant organisms including extended‐spectrum β‐lactamase (ESBL) and quinolone resistant organisms has been reported in recent years relating to TRUS biopsy sepsis.^6^, ^7^ This has resulted in broad‐spectrum carbapenems being used in patients preprocedure and postprocedure.^3^ With the introduction of TPBx, there has been a drastic reduction in the rates of procedure‐related infection.^8^ Compared with TPBx, UTI is 5.4 times more common when TRUS biopsy is performed, with further increased risk if greater than 12 cores are sampled.^2^ A systematic review including 6609 patients found that only 5 (0.076%) patients undergoing TPBx required re‐admission to hospital for treatment of sepsis.^3^ These rates are 40 to 70 times lower than those seen with TRUS biopsy.^3^ This has a significant financial impact on the healthcare system with a reported cost of AUD$7362 per sepsis related admission.^9^

Transperineal template biopsy (TPTB) using a prostate mapping technique has been shown to have a higher detection rate for malignancy, as well as showing a lower rate of postoperative infection and sepsis.^2^ In the past TPTB has been reserved for use in patients on AS undergoing confirmatory biopsy or those with a rising PSA and negative TRUS biopsy.^10^ This has been mainly due to the economic impact it has with relation to the need for general anesthesia (GA), equipment, and increased procedure time. In recent years, technological advances have allowed for the performance of TPBx under local anesthesia (LA).^11^

The PrecisionPoint™ Transperineal Access System (PPTAS) (Corbin Clinical Resources, MD, USA) gained Food and Drug Administration (FDA) approval in 2016.^12^ This system reduces the number of perineal skin punctures to just two as well as removing the requirement for an exaggerated lithotomy position, overall improving patient comfort, and allowing for procedures to be performed under LA only. To our knowledge, there has not been any prospective study directly comparing the outcomes of LA TPBx versus GA TPTB. This is a pilot prospective study that aims to compare the rates of cancer detection, tolerability, as well as patient satisfaction between GA TPTB and LA TPBx using the PPTAS. This study also aims to assess the economic viability of the procedure using surrogate markers for resource consumption.

## 2 METHODS

This is a prospective single center pilot cohort study. The inclusion criteria were all patients undergoing TPBx at our institution from February 2020 to September 2020. Patients who had concurrent procedures such as flexible cystoscopy were excluded to avoid confounding factors affecting the outcomes of this study. Non‐English‐speaking patients, as defined by the hospital admission information, were excluded. This was in relation to the process of reading and understanding the consent and study participant information as well as feasibility for follow‐up phone calls. There were five patients excluded due to language barriers. Patients who met the inclusion and exclusion criteria were given time to read the patient information sheet and were consented for participation in this study. No patients declined to participate in the study. As this is a cohort study, enrolment in the LA versus GA TPBx arm of the study was based on patient preference after informed consent and pre‐operative discussion with patients regarding the risk versus benefits of prostate biopsy performed under LA versus GA. Patients who initially underwent LA TPBx but needed a conversion to GA were not included in the final data analysis but were captured to determine the rate of conversion to GA. As this was a pilot study, sample size calculations were not performed. The study aimed to enroll 80 patients (40 LA PPTAS and 40 GA TPTB).

Prior to the procedure, patients were asked to rate their pain on a numeric rating scale (NRS) numbered 0–10 with 0 being “No pain” and 10 being “Worst pain,” Pain scores were assessed at eight time points: pre‐operatively, LA infiltration, ultrasound probe insertion, biopsy gun firing, leaving recovery, Day 1 (retrospectively during the Day 7 phone call), Day 7, and Day 30. All patients were given a day of surgery questionnaire as well as postoperative phone call questionnaires on Days 7 and 30. A copy of this questionnaire is available in the supporting information.

Clinicopathological data were collected from hospital electronic medical records and included demographic information; age, date of biopsy, prostate‐specific antigen (PSA), comorbidities, digital rectal examination (DRE) findings, multiparametric magnetic resonance imaging (mpMRI) prostate imaging‐reporting and data system (PI‐RADS) score, prostate volume, number of cores taken, and International Society of Urological Pathology (ISUP) histopathological findings. Complications and return to work data were recorded at the time of follow‐up phone call on Days 7 and 30. Ethical approval was granted by the Monash Human Research Ethics Committee on January 29, 2020. Governance approval was obtained from Western Health's Institutional Review Board following this. Informed written consent was obtained from all participants.

Data analysis was performed using *Stata Statistical Software: Release 16* (STATACorp, LLC, TX, USA). Descriptive statistics were used to describe the study groups. Mann–Whitney *U* test was used to compare median values for nonnormally distributed data. Independent *t*‐test was used to compare mean values for normally distributed data. Fisher's exact test of independence was used to determine associations between categorical variables given the small sample size. A two‐sided *p*‐value <0.05 was considered statistically significant.

### 2.1 Biopsy technique

Two transperineal biopsy approaches, LA PPTAS and GA TPTB, were used in this study. All procedures were carried out in the day procedure setting. All patients were prescribed 400‐mcg Tamsulosin for 3 days pre‐operatively and for 7 days postoperatively. All patients were compliant with this pretreatment. Patients received perioperative intravenous (IV) administration of antibiotics: 2‐g cefazolin, 2‐g cefazolin, and 160‐mg gentamicin or 500‐mg ciprofloxacin depending on surgeon preference. Of the 40 LA PPTAS patients, 20 did not receive any antibiotic prophylaxis. All biopsy naïve patients had a prebiopsy mpMRI performed. Tensile tape (Elastoplast) was used to elevate the scrotum away from the perineum. All biopsies had transrectal ultrasound guidance with the BK™ endocavity 8848 ultrasound probe (BK Medical, MA, USA) in the axial and sagittal planes. All patients had a systematic transperineal biopsy regardless of biopsy approach with cognitive‐assisted target sampling if present on prebiopsy mpMRI. The number of biopsies varied depending on prostate size and surgeon preference. All patients were required to void once prior to discharge.

*LA PPTAS*—Patients were placed in the low lithotomy position. LA to the skin of the intended needle entry site was infiltrated with 10 ml of 1% lignocaine with 1:200000 adrenaline. Peri‐prostatic nerve block using up to 15 ml of 1% lignocaine without adrenaline was then performed bilaterally with transrectal ultrasound guidance. LA was given 5 min to take effect. The ultrasound probe with the attached PPTAS device was then reinserted into the rectum and the access needle advanced into the perineum at the previously anesthetized site. The biopsy gun was then introduced through the access needle, and all cores were obtained through the one perineal access point or two perineal access points on each side, depending on prostate size. The threshold for conversion to GA was relatively low to avoid unnecessary patient discomfort. If a patient did not tolerate a DRE on table or did not tolerate the insertion of the local anesthetics needle, they were converted to GA.

*GA TPTB*—Patients were placed in high lithotomy position. Template biopsies utilized the brachytherapy 5‐mm grid/stepper unit (Enovare, PA, USA). Administration of local anesthetic (approximately 20 ml of 0.75% Ropivocaine) to achieve a pudendal nerve block on completion of the procedure was dependent on surgeon preference.

## 3 RESULTS

A total of 40 men underwent GA TPTB and 40 men underwent LA PPTAS during the study period. Comparing the GA TPTB group with the LA PPTAS group, the median age was 63 (interquartile range [IQR] 10.5) versus 63.5 (IQR 10), the mean PSA was 7.60 (±5.02) versus 7.36 (± 4.32), the mean prostate volume was 49.42 (±18.99) versus 51.03 (±17.03), and the median IPSS was 9^13^ versus 7.5 (6.5), respectively. There was no significant difference between the cohorts as to whether patients were having a primary biopsy, repeat biopsy after a negative biopsy, or on active surveillance (*p* = 0.122). The patient characteristics pre‐operatively are summarized in Table 1 and statistically significant differences are highlighted in bold. The only statistically significant difference between the groups at baseline related to PI‐RADS score where the LA PPTAS group had a higher median score (*p* = 0.0326).

**TABLE 1:** Patient characteristics

	GA TPTB (*n* = 40)	LA PPTAS (*n* = 40)	*P*‐value
Age (years)	63 (10.5)	63.5 (10)	0.3123
PSA (ng/ml)	7.60 (±5.02)	7.36 (± 4.32)	0.6204
DRE			0.444
Benign	32.5% (13)	22.5% (9)	
Malignant	20% (8)	15% (6)	
Unknown	47.5% (19)	62.5% (25)	
Prostate volume (ml)	49.42 (±18.99)	51.03 (±17.03)	0.7019
PI‐RADS score	3 (1)	4 (2)	**0.0326**
IPSS (score/35)	9 (14)	7.5 (6.5)	0.4697
ASA classification	2 (0)	2 (0)	0.3437
CCI estimated 10‐year survival	90 (19)	90 (13)	0.1714
Indication for biopsy			0.122
Primary	80% (32)	77.5% (31)	
Previous negative biopsy	0% (0)	10% (4)	
Active surveillance	20% (8)	12.5% (5)	

*Note*: Data are given as median (interquartile range), mean (±standard deviation), or percentage (*n*), unless otherwise stated.

Abbreviations: ASA, American Society of Anesthesiologists; CCI, Charlson comorbidities Index; DRE, digital rectal examination; GA, general anesthetic; IPSS, International Prostate Symptom Score; LA, local anesthetic; ml, milliliter; PI‐RADS, Prostate Imaging‐Recording and Data System; PPTAS, PrecisionPoint™ Transperineal Access System; PSA, prostate‐specific antigen; TPTB, transperineal template biopsy.

At baseline there was a higher median NRS pain score seen in the LA PPTAS (*p* = 0.0080). Using the NRS to evaluate pain postoperatively, the score was not different between the groups on Day 1 (*p* = 0.1783), Day 7 (*p* = 0.2202), or Day 30 (*p* = 0.5686). At the time of leaving the recovery area patients in the GA TPTB group tended to have more pain compared with the LA PPTAS group, but this did not reach the threshold for significance (*p* = 0.0555). When assessing patients return to work following the procedure, there were 38 patients in employment at the time. Of these 81.5% (*n* = 31) returned to work within 7 days of the procedure. Patients who had an LA procedure returned to work significantly faster than those who had a GA (*p* = 0.0459). This is summarized in Table 2 below and statistically significant differences are highlighted in bold.

**TABLE 2:** Outcomes: NRS pain scores, questionnaire, and cancer detection

	GA TPTB (*n* = 40)	LA PPTAS (*n* = 40)	*P*‐value
Pre‐operative NRS score	0 (0)	0 (1.5)	**0.0080**
LA infiltration NRS score	N/A	5 (3)	‐
Probe insertion NRS score	N/A	4 (3)^a^	‐
Biopsy gun fire NRS score	N/A	2 (4)^a^	‐
NRS score leaving recovery	1.5 (3)	0.5 (2)	0.0555
NRS score day 1	1 (3)	0 (2)	0.1783
NRS score day 7	0 (0)	0 (0)	0.2202
NRS score day 30	0 (0)	0 (0)	0.5686
Days until return to work	4 (6.5)^b^	2 (3)^c^	**0.0459**
Number of cores	27 (8)	33 (21.5)	**0.0084**
ISUP grade	1 (1)	2 (1)	0.1601
Cancer detection rate	55% (22)	55% (22)	1.000
Clinically significant cancer	22.5% (9)	35% (14)	0.323

*Note*: Data are given as median (interquartile range), or percentage (*n*) unless otherwise stated.

Abbreviations: GA, general anesthetic; ISUP, International Society of Urological Pathology; LA, local anesthetic; N/A, not applicable; NRS, numeric rating scale; PPTAS, PrecisionPoint™ Transperineal Access System, TPTB, transperineal template biopsy.

Statistically significant differences are highlighted in bold.

^a^ *n* = 20.

^b^ *n* = 24.

^c^ *n* = 14.

In the LA PPTAS group 90% (*n* = 36) reported they would be happy to have this procedure performed again if necessary. Similarly, 97.5% of patients in the GA TPTB group would have the same procedure again (*p* = 0.359). There were two patients (4.8%; *n* = 2/42) excluded from the study who did not tolerate LA infiltration and required conversion to GA biopsy in the study period. Of the 9 patients in the LA group versus the 8 patients in the GA group who had had a previous GA TPTB; 22.2% (*n* = 2) versus 37.5% (*n* = 3) preferred the current procedure, 55.6% (*n* = 5) versus 50% (*n* = 4) felt it was comparable to their previous procedure, and 22.2% (*n* = 2) versus 12.5% (*n* = 1) felt it was worse than their previous procedure.

In the GA TPTB group the overall rate of cancer detection was 55% (*n* = 22). The rate of clinically significant PCa (csPCa) ISUP≥2 was 22.5% (*n* = 9). For patients undergoing a primary biopsy (*n* = 32), the cancer detection rate overall was 53.1% (*n* = 17) and for csPCa was 21.6% (*n* = 7).

In the LA PPTAS group the overall rate of cancer detection was 55% (*n* = 22). The rate of csPCa ISUP≥2 was 35% (*n* = 14). For patients undergoing primary biopsy (*n* = 31), the cancer detection rate overall was 48.3% (*n* = 15) and for csPCa was 35.5% (*n* = 11). There was no statistically significant difference between the two groups when assessing overall cancer detection rate (*p* = 1.000). The median number of cores taken in the GA TPTB cohort was 27 (IQR 8), compared with 33 (IQR 21.5) in the LA PPTAS cohort (*p* = 0.0084).

Acute urinary retention (AUR) requiring catheterization occurred in 5% (*n* = 2) of patients in the GA cohort compared with 2.5% (*n* = 1) of the LA cohort (*p* = 1.000). The episode of AUR in the LA group occurred Day 9 postoperatively and was associated with urosepsis secondary to gram‐negative bacteraemia requiring catheterization and intravenous antimicrobial therapy. The patient presented with urinary retention followed by fever but remained haemodynamically stable through the entire episode and only required ward‐based management.

Culture positive symptomatic UTI occurred in 0% of the GA group and in 5% (*n* = 2) of the LA group (*p* = 0.494). One patient in the GA group was treated with oral antibiotics in the community for dysuria postprocedure; however, his urine sample was sterile. Nonelective re‐admission occurred in 2.5% (*n* = 1) of the GA cohort compared with 5% (*n* = 2) of the LA cohort (*p* = 0.494). All complications that occurred were classified as Clavien‐Dindo ≤2.

There was no difference in procedure specific time between the cohorts (*p* = 0.5292). When anesthetic (local or general) time was included, the GA TPTB cohort had a significantly longer time in theater (*p* < 0.0001). The time the patient was in the theater department from the beginning of the procedure to leaving recovery was significantly shorter in the LA PPTAS group (*p* < 0.0001). Men in the LA PPTAS group spent a significantly shorter period in the recovery area after the procedure (*p* < 0.0001). A summary of the times can be found in Table 3 and statistically significant differences are highlighted in bold.

**TABLE 3:** Surrogate markers for cost analysis

	GA TPTB (*n* = 40)	LA PPTAS (*n* = 20)	*P*‐value
Procedure specific time (min)	21 (12)	20 (12)	0.5292
Procedure time including anesthetic time (min)	39 (12.5)	30.5 (13)	**<0.0001**
Recovery time (min)	51 (16.5)	18.5 (14)	**<0.0001**
Total time (min)	93 (19.5)	54 (17)	**<0.0001**

*Note*: Data are given as median (interquartile range).

Abbreviations: GA, general anesthetic; LA, local anesthetic; PPTAS, PrecisionPoint™ Transperineal Access System; TPTB, transperineal template biopsy.

Statistically significant differences are highlighted in bold.

## 4 DISCUSSION

In recent years there has been a shift towards the TP route for prostate biopsies. Many centers have moved to TPBx to avoid complications related to fecal contamination, at the cost of utilizing theater time for TPBx under GA. The requirement for sedation or GA relates to patient tolerability due to the exaggerated positioning of the patient required to place a stepper and grid, as well as the need for a skin puncture per biopsy taken. Advancements have recently been made in device technologies to allow for TPBx to be performed under LA using the PPTAS and other devices.^11^, ^13^, ^14^, ^15^ This method not only benefits the patient by eliminating the risk of undergoing GA but also has a potential economic impact on the healthcare system given the reduced resources required.

This study demonstrates that LA TPBx is well tolerated with 95.2% of patients able to complete the procedure. The 4.8% rate of conversion to GA is consistent with previous studies on LA TPBx.^16^, ^17^ Kum et al. previously reported LA injection to be the most painful part of the LA TPBx procedure.^16^ The authors in this study had a similar experience, and this present study showed that the pain score during LA injection to be manageable with a median of 5 (IQR 3) on the NRS. The pain score recorded for ultrasound probe insertion (4 [IQR 2]) and the firing of the biopsy gun (2 [IQR 4]) also appear tolerable. Relating to the previous studies reviewing LA PPTAS, the paper published by Meyer et al. in 2018 was retrospective in nature and did not objectively assess patient's perioperative pain with a validated system.^11^ Unlike the present study where an NRS of 0–10 was used, Kum et al. and Gorin et al. assessed pain using a Visual Analog Scale (VAS) or the Wong–Baker Scale, respectively.^16^, ^17^ All of these papers concluded that LA PPTAS was tolerable for patients.

Pain scores on leaving the recovery area trended towards being higher in the GA TPTB group compared with the LA PPTAS group (*p* = 0.0555). The use of a pudendal nerve block post operatively in the GA TPTB group was at the discretion of the operating surgeon and therefore was not administered to all patients. This may contribute to the pain score postoperatively in the GA TPTB group. The GA TPBx patients also had a higher number of puncture sites due to the nature of the procedure which may have contributed to their pain scores. In the follow‐up questionnaire 90% (*n* = 36) of LA patients reported, they would be satisfied to have the same procedure again where they were to require a repeat biopsy. This compares to 97.5% (*n* = 39) of the GA cohort (*p* = 0.359).

It has been well documented that the TP route of biopsy has a superior cancer detection rate compared with TRBx.^4^, ^18^ In a previous local audit reviewing 439 biopsy naïve patients, it was determined that the overall cancer detection rate in the first‐biopsy setting between 2017 and 2018 was 54.7%.^19^ In the literature there have been many hypotheses as to the root of this with some suggesting the advent of mpMRI prior to biopsy allowing for targeted biopsy and others noting the superior sampling of the anterior zone of the prostate.^20^, ^21^ In the current study the cancer detection rates with LA PPTAS (55%) and GA TPTB (55%) have been consistent with other studies in the literature including Kum et al. (76.3%), Gorin et al. (83.2%), and Meyer et al. (41.9%).^11^, ^16^, ^17^ Unfortunately, the relatively high cancer detection rates in some of these studies are difficult to interpret given there was no control group in the study. The present study is therefore the first to demonstrate that PPTAS cancer detection rates are not inferior to TPTB (*p* = 1.000). A recently published study looking at 174 standard grid‐based biopsy patients compared with 304 freehand biopsy patients has shown an equivalent cancer detection rate between both techniques.^22^

In the first 7 days following the procedure, there were no documented new infections in either group, indicating that there was no introduction of bacteria via the needle biopsy. This is consistent with previous literature and supports the adoption of the TP route rather than the TR route as a safer method of biopsy by avoiding fecal contamination.^2^, ^3^, ^8^ Unfortunately, there was an infectious complication relating to a patient suffering from AUR on day 9 following the operation. This patient was re‐admitted to hospital and treated with antimicrobial therapy for gram‐negative bacteraemia. The patient had a single recorded fever, did not exhibit any hemodynamic changes, nor require any inotropic support. The patient recovered uneventfully. Although this is secondary to the AUR and not related to the introduction of bacteria into the prostate via the needle biopsy, it remains an infectious complication following the procedure. The second patient recorded as an infection was symptomatic for a UTI prior to biopsy and was commenced on antimicrobial therapy at the time of biopsy. However, the overall rate of infection remains low at 5% (*n* = 2) and is in keeping with other PPTAS studies.^16^, ^17^ Interestingly, in the 20 LA PPTAS patients who did not receive antimicrobial prophylaxis. there were no new infective complications recorded up to 30 days postoperatively. There has been concern in recent years in relation to antibiotic resistance related to the use of perioperative antimicrobial prophylaxis and treatment.^3^, ^7^ The practice in previous literature related to the PPTAS device varies widely in terms of antibiotic prophylaxis, as expected given the lack of clear guidelines. All studies demonstrate a low or nil rate of infection whether prophylaxis with gentamicin is given or interestingly, whether no antimicrobial is given at all. These results are encouraging and may suggest that the use of the PPTAS device contributes to a reduced infective risk compared with TRUS biopsy. Clear guidelines on antimicrobial use perioperatively are needed.

The rate of AUR requiring catheterization in the present study was 5% (*n* = 2) in the GA TPTB group which was not statistically different to the 2.5% (*n* = 1) in the LA PPTAS group (*p* = 1.000). In line with current local guidelines, patients were given 400‐mcg Tamsulosin for 3 days pre‐operatively and this was continued for a further 7 days postoperatively. Compliance with this pretreatment was recorded, and all patients were compliant. Compared with the previous literature on PPTAS, our rate is consistent with Meyer et al. (4.7%), Kum et al. (0.6%), and Gorin et al. (1.1%). Urkmez et al. have assessed 304 patients following a freehand biopsy and reported an AUR rate of just 1%.^22^ At its inception TPBx‐related AUR was a cause for concern; however, the widespread use of α‐blockade pre‐operatively has mitigated that risk.

Prostate biopsy techniques are fast evolving, especially when examining TP techniques. Since TPBx was first introduced with the standard template biopsy, some centers have since moved to exclusively performing MRI‐targeted biopsies.^23^, ^24^, ^25^ The standard of care is constantly changing since the advent of mpMRI prior to biopsy, and the debate continues as to the most effective and efficient biopsy strategy.^26^ Without the requirement of GA, and by proxy removing the requirement for an anesthetist to be present, amounts to a saving $174.20/hour of theater time (based on specialist year 5/9, FPPCOA 17.6 + hours/week January 2020). Also removed from the cost of the procedure is an anesthetic nurse, saving on average $33.66/hour of theater time (based on registered nurse, level 3/5 full‐time or part‐time July 2020). Anesthetic equipment including ventilation masks, tubing, and cannulas, as well as induction and maintenance of anesthesia medications, are also saved. Surgical equipment omitted are the cost of the CIVCO™ stepper, workstation stabilizer, transportation stand, endocavity balloon, disposable template grid, and grid adapters. However, this is weighed up against the additional cost of the disposable PPTAS device which is $350 per device.

Consumption of less theater and recovery times allows the theater resources to be more efficiently utilized to care for more patients. This is important, especially during the COVID‐19 pandemic, with a scarcity of theater resources available to manage the ever‐expanding waiting list. Furthermore, utilization of theater and recovery time can be considered a surrogate marker for cost. Although procedure specific time was similar between the groups (*p* = 0.5292), the overall time spent by the patient in the theater including administration of anesthesia was significantly longer in the GA TPTB group (*p* < 0.001). Given the PPTAS device is new to the surgeons involved, it is anticipated that the procedure time will decrease with increasing experience of the device. In further studies, precise and comprehensive costing per patient will be obtained.

### 4.1 Limitations

This study has a relatively small sample size due to the nature of it being a pilot study. As such, the study may be underpowered to detect differences in outcomes such as cancer detection rates and complication rates. However, even at this small number, this study detected a difference in the postoperative pain score, as well as a significant difference in use of theater resources between the two techniques.

Participants were not randomly allocated to the method of biopsy and instead chose themselves whether to have GA or LA. The selection bias may have affected outcomes such as pain scores and conversion rate to GA. However, this is representative of real‐life scenario where all patients would require informed consent prior to proceeding with any procedure. Selection bias through the exclusion of non‐English speaking patients has also been noted; however, given the small number of patients this excluded, it is not thought that this is significant. Furthermore, the authors of this study aim to conduct further prospective randomized controlled trials in the future based on the results of this pilot study, which will endeavor to control for this possible bias.

Lack of standardization of technique due to multiple surgeons being involved may be another confounding factor. There are no clear guidelines in relation to the use of antimicrobial agents pre‐operatively, the use of pudendal nerve block postoperatively, or the number of cores required, and each varies widely depending on surgeon preference. These factors may have influenced some outcome measures such as cancer detection, pain scores, and complication rates. However, this also represents real‐world scenario where surgeons vary slightly in their techniques and practices.

## 5 CONCLUSION

This prospective pilot cohort study demonstrates that LA TPBx is safe, effective, and tolerable. The pain score given for the maximum discomfort point of the procedure was a median of 5/10. The total operative time including anesthesia was shorter in the LA PPTAS group, and patients spent a shorter time in recovery after the LA procedure. This reduction in time saves theater resources and will allow a higher number of patients to be biopsied at any given time. Given these findings, a precise cost comparison of these procedures is required to investigate financial viability, especially given the aging population and the foreseen increase in the prevalence of PCa. Further studies to confirm the findings of this pilot study are warranted.

## CONFLICT OF INTERESTS (ICMJE DISCLOSURE)

We confirm that this paper has not been published or submitted for publication elsewhere and that all authors have contributed significantly and are all in agreement with the content of the manuscript. The following apply to all listed authors:

- All support for the present manuscript (e.g., funding, provision of study materials, medical writing, and article processing charges): none
- Grants or contracts from any entity: none
- Royalties of licenses: none
- Consulting fees: none
- Payment or honoraria for lectures, presentations, speakers' bureaus, manuscript writing, or educational events: none
- Payment for expert testimony: none
- Support for attending meetings and/or travel: none
- Patents planned, issued, or pending: none
- Participation on a data safety monitoring board or advisory board: none
- Leadership or fiduciary role in other board, society, committee or advocacy group, paid or unpaid: none
- Stock or stock options: none
- Receipt of equipment, materials, drugs, medical writing, gifts, or other services: none
- Other financial or nonfinancial interests: none

## Supporting information


**Data S1.** Supporting Information
