# Supplementary material for: Local versus general anesthesia transperineal prostate biopsy: Tolerability, cancer detection, and complications
Source: BJUI Compass. 2021 Sep 10;2(6):428–35. doi: 10.1002/bco2.106 (PMC8988812; doi:10.1002/bco2.106)
Supplement: Supplementary file 1 — Data S1. Supporting Information [file BCO2-2-428-s001.pdf]

Western Health
MonashHealth

Study ID: ________

Questionnaire
PrecisionPoint^{TM} Prostate Biopsy

Date: __/__/__

International Prostate Symptoms Score (IPSS) – Pre-operative

	Not at all	Less than 1 time in 5	Less than half the time	About half the time	More than half the time	Almost always	Your score
**Incomplete emptying** Over the past month, how often have you had a sensation of not emptying your bladder completely after you finish urinating?	0	1	2	3	4	5	
**Frequency** Over the past month, how often have you had to urinate again less than two hours after you finished urinating?	0	1	2	3	4	5	
**Intermittency** Over the past month, how often have you found you stopped and started again several times when you urinated?	0	1	2	3	4	5	
**Urgency** Over the last month, how difficult have you found it to postpone urination?	0	1	2	3	4	5	
**Weak stream** Over the past month, how often have you had a weak urinary stream?	0	1	2	3	4	5	
**Straining** Over the past month, how often have you had to push or strain to begin urination?	0	1	2	3	4	5	

	None	1 time	2 times	3 times	4 times	5 times or more	Your score
**Nocturia** Over the past month, many times did you most typically get up to urinate from the time you went to bed until the time you got up in the morning?	0	1	2	3	4	5	

Add Your scores and write total in the box to the right.	

Total score: 0-7 Mildly symptomatic; 8-19 moderately symptomatic; 20-35 severely symptomatic.

Quality of life due to urinary symptoms	Delighted	Pleased	Mostly satisfied	Mixed: Equally satisfied / dissatisfied	Mostly dissatisfied	Unhappy	Terrible
If you were to spend the rest of your life with your urinary condition the way it is now, how would you feel about that?	0	1	2	3	4	5	6

Study ID: ________

Questionnaire
PrecisionPoint™ Prostate Biopsy

Date: __/__/__

Pain Score Pre-Operative

No Pain					Moderate Pain					Worst Pain
0	1	2	3	4	5	6	7	8	9	10

Pain Score LA Infiltration

No Pain					Moderate Pain					Worst Pain
0	1	2	3	4	5	6	7	8	9	10

Pain Score Ultrasound Probe Insertion

No Pain					Moderate Pain					Worst Pain
0	1	2	3	4	5	6	7	8	9	10

Pain Score Biopsy Gun Fire

No Pain					Moderate Pain					Worst Pain
0	1	2	3	4	5	6	7	8	9	10

Pain Score Immediately Post-Operative

No Pain					Moderate Pain					Worst Pain
0	1	2	3	4	5	6	7	8	9	10

Study ID: ________

Questionnaire
PrecisionPoint™ Prostate Biopsy

Date: __/__/__

Follow- Up Phone Call Day 7 and Day 30 post-op

Patient ID Confirmed with 3-point check ☐

Q1a. Have you been able to return to work?

Y / N

Q1b. If "no" then what has impeded this?

____

Q1c. If "yes" when did you return to work?

____

Q2. On a scale of 0-10 with "0" being no pain at all, and "10" being the worst pain, how much pain do you currently have?

0 1 2 3 4 5 6 7 8 9 10

Q3. Did you have any urinary tract infections in the last 7/30 days

Q4. Did you have any inability to pass urine in the last 7/30 days

Q5. Were you re-admitted to hospital in the last 7/30 days

Q6. If you were to require a further prostate biopsy in the future would you have this procedure again?

Y / N

Q7a. Is this your first prostate biopsy?

Y / N

Q7b. How was your previous biopsy undertaken?

General anaesthesia / Local anaesthesia

Q7c. Would you describe your most recent procedure as better, comparable or worse in comparison to your previous biopsy?

Better/Comparable/Worse
